# Contribution to the study of the Afrotropical Pyraustinae Meyrick, 1890 (Lepidoptera, Crambidae): Taxonomic and distributional updates on five species with new country records

**DOI:** 10.3897/BDJ.13.e161293

**Published:** 2025-07-30

**Authors:** Michael Seizmair, Alrabea Ishag, Hathal Mohammed Al Dhafer

**Affiliations:** 1 SNSB Bavarian State Collection of Zoology Munich (Independent Associated Scientist), Munich, Germany SNSB Bavarian State Collection of Zoology Munich (Independent Associated Scientist) Munich Germany; 2 King Saud University Museum of Arthropods, Department of Plant Protection, College of Food and Agriculture Sciences, King Saud University, Riyadh, Saudi Arabia King Saud University Museum of Arthropods, Department of Plant Protection, College of Food and Agriculture Sciences, King Saud University Riyadh Saudi Arabia; 3 King Saud University, College of Food and Agriculture Sciences, Riyadh, Saudi Arabia King Saud University, College of Food and Agriculture Sciences Riyadh Saudi Arabia

**Keywords:** Pyraloidea, Pyraustini, Euclastini, morphology, distribution, fauna of the Arabian Peninsula, new combination

## Abstract

**Background:**

The Pyraustinae Meyrick, 1890 form the fifth most diverse subfamily in the Pyraloidea, comprising 1270 described species distributed over 173 genera. For the Afrotropical zone, a total of 160 species have become known to date. Faunistic studies on the subfamily for the Afrotropical zone have been to date regionally confined on the southern parts of Africa, including South Africa, the Mascarene and Malagasy islands. The other parts of sub-Saharan Africa have remained understudied. This results in still unexplored distribution patterns of the Afrotropical Pyraustinae. The Afrotropical zone reaches its northern boundaries in the southern parts of the Arabian Peninsula which include the southern part of Oman (Province Dhofar), Yemen and the south-western parts of Saudi Arabia. For the Pyraustinae, the Arabian Peninsula has been little explored to date. No more than 20 species distributed over 13 genera have become known.

**New information:**

Taxonomic and distributional updates are presented on five species of the Pyraustinae (Lepidoptera, Crambidae), based on material from East and Central Africa and from the tropical parts of the Arabian Peninsula. *Ecpyrrhorrhoediatoma* (Hampson, 1913) and *Psammotishaematidea* (Hampson, 1913) are re-described, with the male and female genitalia described and figured for the first time. A new combination *Paschiodespostmediofusalis* (Seizmair, 2022) **comb. n.** from *Pyrausta* Hübner, 1825 is introduced, with the diagnostic characters differentiating the species from the other species of the Afrotropical genus *Paschiodes* Hampson, 1913 being listed. *Psammotishaematidea* (Hampson, 1913), *Ecpyrrhorrhoediatoma* (Hampson, 1913) and *Euclastawarreni* Distant, 1892 are reported as new to the Arabian Peninsula. *Psammotishaematidea* (Hampson, 1913) is furthermore reported as new to Kenya and Cameroon. *Paschiodespostmediofusalis* (Seizmair, 2022) **comb. n.** and *Euclastavarii* Popescu-Gori & Constantinescu, 1973 are reported as new to Saudi Arabia. For each of the taxa presented, external and internal diagnostic characters are listed and illustrated.

## Introduction

The Pyraustinae Meyrick, 1890 form the fifth most diverse subfamily in the Pyraloidea, comprising 1270 described species distributed over 173 genera (Landry 2015, Mally et al. 2019 and Nuss et al. 2024). Mally et al. (2019) divide the subfamily into three tribes. A vast majority of the assigned genera is included in the Pyraustini Meyrick, 1890, with no more than five genera attributed to the Portentomorphini Amsel, 1856 and only two valid genera included in the Euclastini Popescu-Gori & Constantinescu, 1977 (Mally et al. 2019 and Nuss et al. 2024). Some 53 % of the genera in the subfamily have remained unassigned to date (Nuss et al. 2024). The subfamily has a world-wide distribution.

For the Afrotropical zone, 160 species have become known to date (De Prins and De Prins 2025). The vast majority of the studies on the African Pyraustinae date to historical work from the beginning of the last century including studies by Hampson (1913a), Hampson (1913b), Hampson (1918a)and Hampson (1918b) . Recent faunistic studies on the subfamily have been to date restricted to the southern parts of Africa including South Africa, the Malagasy and the Mascarene islands (Vari et al. 2002, Shaffer and Munroe 2007and Guillermet 2009). The other parts of sub-Saharan Africa have still been little explored for the subfamily, with comprehensive updated faunistic checklists still missing. Poltavsky et al. (2019) listed no more than six species of the Pyraustinae in their Pyraloidea study on Liberia. Rougeot (1984) mentioned only three species of the subfamily in his Heterocera study on Ethiopia. Further important partial taxonomic revisions with descriptions of new genera considered as endemic include studies by Shaffer and Munroe (2007), Maes (2000), Maes (2001a), Maes (2001b), Maes (2001c), Maes (2002), Maes (2005), Maes (2006a), Maes (2006b), Maes (2009), Maes (2014) and Maes (2023) .

The Afrotropical zone reaches its northern boundaries in the southern parts of the Arabian Peninsula which include the southern part of Oman (Province Dhofar), Yemen and the south-western parts of Saudi Arabia. The potential of this region both for African species reaching their northernmost distribution boundaries and for endemic species has been shown for the Noctuoidea Latreille, 1809 and Geometridae Stephens, 1829 in studies including Hausmann (2006) andHacker (2016). For the Pyraloidea, however, the Arabian Peninsula has been little explored to date. For the Pyraustinae, no more than 20 species distributed over 13 genera have become known in historical studies dating to Walsingham and Hampson (1896) and in recent studies including Pelham-Clinton (1977), Asselbergs (2008), Seizmair (2021), Seizmair (2022), Seizmair (2023a) and Seizmair (2023b).

In this study, the two little known species *Psammotishaematidea* (Hampson, 1913) and *Ecpyrrhorrhoediatoma* (Hampson, 1913) are re-described and new country records from the Arabian Peninsula and the sub-Saharan African mainland are provided. Further distributional updates, based on new country records from Saudi Arabia, are provided for *Euclastawarreni* Distant, 1892 and *Euclastavarii* Popescu-Gori & Constantinescu, 1973. A new combination *Paschiodespostmediofusalis* (Seizmair, 2022) **comb. n.** from *Pyrausta* Schrank, 1802 is introduced. The species is reported as new to the fauna of Saudi Arabia.

## Materials and methods

### Sampling

The material presented in this study is part of samples collected by the authors in Saudi Arabia in the surroundings of Rayda (Province Asir) and of Shada (Al Baha) in research projects of the KSMA in the years 2014 and 2015, in the surroundings of Abha (Province Asir) and in the Fayfa Mts. (Province Jizan) in the years 2022 – 2024 and in the Province Dhofar of the Sultanate of Oman in 2019. Further distributional data from East and Central Africa – Kenya, Cameroon – in the period 1997–2019, were made available to the authors from the ABSRC (Koen V. N. Maes, Wetteren, Belgium). Details on the collection data are given in the material examined sections. The specimens were captured at night by means of light traps equipped with UV tubes and UV power-LEDs. The UV power-LEDS cover a wave spectrum of 365 nm – 385 nm (LepiLED, Nichia, Tokushima, Japan; EntoLED, Starlight, Weissenburg, Germany).

### Preparation, dissection and digital image processing

The adults were photographed after relaxation and subsequent preparation with a CANON EOS M6 Mark II under a MP-E-65 mm zoom or with the LEICA imaging system of the KSMA. For examining the genitalia, slide-mounting techniques were applied on the specimens according to the protocol described by Robinson (1976). The genitalia were prepared with Motic (SMZ-171) and Meiji stereomicroscopes. The slides were photographed with a ToupCam c-mount camera (ToupTek Inc., Zhejiang, China). Image post-processing were was performed by means of Adobe Photoshop PS, Version 23.5.2.

### Morphological analyses and comparisons

Analyses of wing pattern characters and morphological structures in the specimens from the KSMA and CMS were performed on the images. Structural ratios were calculated on the images by means of the imaging software ToupView, Version 4.12 (ToupTek Inc., Zhejiang, China).The specimens were determined by external and internal morphological characters as listed in Popescu-Gorj and Constantinescu (1973), Popescu-Gorj and Constantinescu (1977), Slamka (2013), Maes (2000) and Seizmair (2022). The specimens of the genera *Psammotis* Hübner, 1825 and *Ecpyrrhorrhoe* Hübner, 1825 were compared with relevant type material (det. Koen V. N. Maes).

### Terminology and abbreviations

The descriptions of external and internal character states follows the terminology in Maes (1995), Mally et al. (2019) and Nuss et al. (2024). Descriptions of the wing venation follow the terminology in Shaffer and Munroe (2007).

Abbreviations of depositories:

- ABSRC = AgroBioSys International Reference Collection, Wetteren, Belgium
- CMS = private research collection Michael Seizmair, Gröbenzell, Germany
- KSMA = King Saud University Museum of Arthropods, Riyadh, Saudi Arabia
- RMCA = Royal Museum for Central Africa, Tervuren, Belgium

Other abbreviations:

- slide no = slide number
- specimen no = specimen number
- n = cardinality / length of a sample

## Taxon treatments

### Psammotis haematidea

(Hampson, 1913)

5806E187-5794-5ECD-9723-A7DE14659A78

*Pyraustahaematidea* Hampson, 1913. Hampson (1913b): 31-32. Type locality: Tanzania, Ruaha R[iver]

#### Materials

- **Type status:** Other material. **Occurrence:** catalogNumber: ♂24GP028; recordedBy: M. Seizmair, A. Ishag; individualCount: 1; sex: male; lifeStage: adult; disposition: CMS; occurrenceID: 80000C12-B756-58A8-A7BB-F2A7705E326E; **Location:** country: Saudi Arabia; stateProvince: Jizan; locality: Fayfa Mts., Al Khashah; verbatimElevation: 640 m; verbatimLatitude: 17°14' 30.3684"; verbatimLongitude: 43° 3' 41.0178"; **Event:** samplingEffort: UV- Light; eventDate: 10-XI-2023
- **Type status:** Other material. **Occurrence:** catalogNumber: ♀24GP027; recordedBy: M. Seizmair, A. Ishag; individualCount: 1; sex: female; lifeStage: adult; disposition: CMS; occurrenceID: 63A0C80B-282D-54F8-9142-561412D6ADCA; **Location:** country: Saudi Arabia; stateProvince: Jizan; locality: Fayfa Mts., Al Khashah; verbatimElevation: 640 m; verbatimLatitude: 17°14' 30.3684"; verbatimLongitude: 43° 3'41.0178"; **Event:** samplingEffort: UV- Light; eventDate: 13-XI-2023
- **Type status:** Other material. **Occurrence:** catalogNumber: ABSRC1000599 (slide no ♂1854); recordedBy: K. Maes; individualCount: 5; sex: male; lifeStage: adult; disposition: ABSCR; otherCatalogNumbers: ABSRC1000600 (slide no ♂1855), ABSRC1000683, ABSRC1004998, ABSRC1004999; occurrenceID: 4D32E076-B86B-5077-9DAB-2FEA798452E6; **Location:** country: Cameroon; locality: Center Region, Savannah-Rainforest edge, near Magong, SE of Yoko; verbatimElevation: 732 m; verbatimLatitude: N 05°23’38.5''; verbatimLongitude: 012°30’48.4”; **Event:** samplingEffort: Black/MV lights; eventDate: 04 -11-VI-2019
- **Type status:** Other material. **Occurrence:** catalogNumber: ABSRC1000653; recordedBy: K. Maes; individualCount: 8; sex: male; lifeStage: adult; disposition: ABSCR; otherCatalogNumbers: ABSRC1000654, ABSRC1000656 - ABSRC1000661; occurrenceID: 77BC4A7C-BD96-530F-A0D9-40D8EBD06149; **Location:** country: Cameroon; locality: Center Region, Savannah-Rainforest edge, near Magong, SE of Yoko; verbatimElevation: 732 m; verbatimLatitude: N 05°23’38.5''; verbatimLongitude: 012°30’48.4”; **Event:** samplingEffort: Black/MV lights; eventDate: 05 -11-VI-2019
- **Type status:** Other material. **Occurrence:** catalogNumber: ABSRC1005053; recordedBy: K. Maes; individualCount: 1; sex: male; lifeStage: adult; disposition: ABSCR; occurrenceID: 0F79783F-1588-53AA-87B8-9D94170255D6; **Location:** country: Cameroon; locality: Center Prov. Batchenga; **Event:** eventDate: 19-VI-1993
- **Type status:** Other material. **Occurrence:** catalogNumber: ABSRC1005051; recordedBy: K. Maes; individualCount: 3; sex: male; lifeStage: adult; disposition: ABSCR; otherCatalogNumbers: ABSRC1005055, ABSRC1005062 (slide no ♂918); occurrenceID: 40E8B73C-993C-5D5C-88DB-001C57F87CC3; **Location:** country: Kenya; locality: Rift Valley, Marich Pass Field Station area; verbatimElevation: 950 m; verbatimLatitude: 01°32’14”; verbatimLongitude: 35°27’26”; **Event:** samplingEffort: Mercury Vapor Light; eventDate: 26- 29-VII-1999
- **Type status:** Other material. **Occurrence:** catalogNumber: ABSRC1005052; recordedBy: K. Maes; individualCount: 1; sex: male; lifeStage: adult; disposition: ABSCR; occurrenceID: FA118907-92E9-5485-93A3-6B241F9BE5E3; **Location:** country: Kenya; locality: Eastern, Meru N.P. Bwatherongi Camp Site; verbatimElevation: 600 m; verbatimLatitude: 00°09’51.8”; verbatimLongitude: 38°12’32.3”; **Event:** samplingEffort: Black/MV lights; eventDate: 04-XII-2002
- **Type status:** Other material. **Occurrence:** catalogNumber: ABSRC1005054; recordedBy: K. Maes; individualCount: 5; sex: male; lifeStage: adult; disposition: ABSCR; otherCatalogNumbers: ABSRC1005058, ABSRC1005059, ABSRC1005060, ABSRC1005061 (slide no ♂1438); occurrenceID: 7A95F11A-C6EC-54F3-94B6-C7A52C171DFF; **Location:** country: Kenya; locality: Kakamega Rainforest Edge “Quarry”; verbatimElevation: 1800 m; verbatimLatitude: 0° 10' 12"; verbatimLongitude: 34° 28' 11.9994"; **Event:** samplingEffort: Mercury Vapor Light; eventDate: 07-V-1997
- **Type status:** Other material. **Occurrence:** catalogNumber: ABSRC1005056; recordedBy: K. Maes; individualCount: 5; sex: male, female; lifeStage: adult; disposition: ABSCR; otherCatalogNumbers: ABSRC1005057, ABSRC1005064, ABSRC1005065, ABSRC1005066; occurrenceID: DAC3EB3E-0FD7-55EF-9A88-2F01E28993B4; **Location:** country: Kenya; locality: Rift Valley, Samburu Nat.Res.near Uaso Nyiro river, Intrepid Camp; verbatimElevation: 910 m; verbatimLatitude: 0°34’34,8”; verbatimLongitude: 37°39’36”; **Event:** samplingEffort: Black/MV lights; eventDate: 13-14-XII-2002
- **Type status:** Other material. **Occurrence:** catalogNumber: ABSRC1005063 (slide no ♀919); recordedBy: K. Maes; individualCount: 1; sex: female; lifeStage: adult; disposition: ABSCR; occurrenceID: CBFB12C9-E8DD-5DB1-A759-19496959549C; **Location:** country: Kenya; locality: Coast, Rukinga Ranch, Sagana Dam; verbatimElevation: 462 m; verbatimLatitude: 03°51’09.6”; verbatimLongitude: 38°50’39.7”; **Event:** samplingEffort: Black/MV lights; eventDate: 30-XII-2000

#### Description

##### External characters

**Re-description**- Wing span: 10.0 mm (male, n = 1), 10.7 mm (female, n = 1), fore-wing length: 5.2 mm (male, n = 1), 6.3 mm (female, n = 1). Head: Antenna yellowish in the flagellum, greyish-white in the ciliae in both sexes. Vertex and frons with reddish and yellowish scales. Labial palpus with an extended patch of elongate shining white scales ventrally in the first segment, scaling of the second and third segments brownish-yellowish interspersed with greyish and reddish scales, length relative to the diameter of the eye 1.7 (n = 1, male). Maxillary palpus obliquely upturned, with brownish-yellowish scaling, length relative to the length of the labial palpus 0.2 (n = 1, male). Thorax: Scaling of the dorsum reddish-brown interspersed with yellowish scales, scaling of the venter greyish-white in both sexes. Fore-wing upper side ground reddish, antemedial, postmedial and subterminal lines yellow. Postmedial line with a right-angled inward turn at CuA1, then running upwards towards the cell, with another right-angled turn at CuA1 towards the anal border. Subterminal line developing from the costa, interrupted at R5, continuing strongly tapered at CuA1 and terminating at the anal border. Costa with yellow scales between the medial area and the apex. Fringe yellow, long-scaled. Hind-wing ground concolorous with the fore-wing ground. Postmedial line concolorous with the fore-wing postmedial line, strongly broadened, w-shaped. Fringe yellow, with elongate reddish scales at the anal border. There are no intersexual differences in the wing maculation. Legs greyish-brown in the femur, greyish-white in the tibia. Abdomen: Dorsum with reddish scaling, in the male with a yellowish-grey anal tuft, venter greyish-white (Hampson (1913b), Figs 1a, 2a).

##### Male genitalia

Uncus 1.8 times longer than wide (n = 1), apex pointed, with very short chaetae dorso-laterally, ventrally with two overlapping flanges of rhomboid shape and with anteriad-directed process. Transtilla widely separated, transtillum inferior well developed. Valva strongly broadened in the anterior half, posteriorly constricted, apex medially rounded, costa and ventral border parallel, basal costa inflated, base of the editum anteriorly thumb-shaped, posteriorly bifid with acuminate ends, sella with a posterior cup-shaped extension, anterior end hook-shaped. Sacculus post-basally dilated, with a sclerotised dorsal ridge. Arms of the juxta slender, strongly elongated, acuminate, length of the split relative to the total length 0.6. Vinculum u-shaped, short, 1.8 times wider than long (n = 1). Phallus cylindrical, constant in width. Vesica with two spiculose patches and one thorn-shaped, slightly curved cornutus (Figs 3a, 4a).

##### Female genitalia

Length of the posterior apophysis relative to the anterior apophysis 0.7 (n = 1). Anterior apophysis with a subtriangular-shaped medial projection. Posterior ductus bursae sclerotised. Colliculum short. Antrum sclerotised, elongated. Rhomboid signum with longitudinal and transversal sclerites, ratio length of longitudinal/length of transversal axis 0.6 (n = 1), lateral processes short, rounded. Bursa with further sclerotisation at the appendix (Fig. 5a).

#### Diagnosis

The species is externally easily differentiated from the other congeners by the presence of an angulated yellowish postmedial line in the fore-wing and by the presence of a wedge-shaped hind-wing postmedial line. The items in the wing maculation as mentioned in the re-description above are in conformance with the original description in Hampson (1913b). From the externally closest *Psammotisrubrilinearis* Seizmair, 2023, the species is furthermore differentiated in the male genitalia by the presence of a pair of processed flanges in the uncus, by the shape of the editum basis with a posterior bifurcation, by the shape of the sella, the anterior end of which is curved towards the ventral border of the valva and by the elongated arms of the bifid juxta.

#### Distribution

Afrotropical. To date only known from the type locality in Tanzania (De Prins and De Prins 2025). **New to the Arabian Peninsula, to Cameroon and to Kenya.**

### Ecpyrrhorrhoe diatoma

(Hampson, 1913)

3D426F47-0FB1-59ED-BB7F-BA9DF17B4D65

*Pyraustadiatoma* Hampson, 1913. Hampson (1913b): 35. Type locality: South Africa, KwaZulu-Natal [Natal].

#### Materials

- **Type status:** Other material. **Occurrence:** catalogNumber: 24GP032; recordedBy: M. Seizmair, A. Ishag; individualCount: 3; sex: male; lifeStage: adult; disposition: CMS; otherCatalogNumbers: 24GP033, 24GP034; occurrenceID: E7EB0965-0D83-5943-A65F-7EFFBDE684E5; **Location:** country: Saudi Arabia; stateProvince: Jizan; locality: Fayfa Mts., Al Kasha; verbatimElevation: 640 m; verbatimLatitude: 17°14' 30.3684"; verbatimLongitude: 43° 3' 41.0178"; **Event:** samplingEffort: UV Light; eventDate: 11-XI-2023
- **Type status:** Other material. **Occurrence:** catalogNumber: 22GP066; recordedBy: M. Seizmair; individualCount: 1; sex: female; lifeStage: adult; disposition: CMS; occurrenceID: 78887F27-4642-543E-A88E-CD26A0421C9A; **Location:** country: Saudi Arabia; stateProvince: Jizan; locality: 5km NW Fayfa; verbatimElevation: 600 m; verbatimLatitude: 17°15'40.24"; verbatimLongitude: 43°4'25.16"; **Event:** samplingEffort: UV Light; eventDate: 22-IX-2022
- **Type status:** Other material. **Occurrence:** catalogNumber: RMCA ENT 000007733; recordedBy: U.Dall'Asta; individualCount: 1; sex: male; lifeStage: adult; disposition: RMCA; occurrenceID: EC006F61-DEA7-52EE-AABC-B21BD1350EB6; **Location:** country: Kenya; locality: Kakamega Forest; verbatimElevation: 1580 m; verbatimLatitude: 00°19'; verbatimLongitude: 34°52'; **Event:** eventDate: 17-X-2001
- **Type status:** Other material. **Occurrence:** catalogNumber: ABSRC1004140; recordedBy: A.& I.Sharp; individualCount: 1; sex: male; lifeStage: larva; disposition: ABSRC; occurrenceID: BFFF638B-74B5-52D5-932D-9C6EF239AE47; **Location:** country: South Africa; locality: Casketts farm, Hoedspruit; **Event:** eventDate: 15-XII-2012
- **Type status:** Other material. **Occurrence:** catalogNumber: ABSRC1005014; recordedBy: K. Maes; individualCount: 20; sex: female; lifeStage: adult; disposition: ABSRC; otherCatalogNumbers: ABSRC1005014, ABSRC1005015, ABSRC1005016, ABSRC1005017, ABSRC1005021, ABSRC1005018, ABSRC1005024, ABSRC1005023, ABSRC1005025, ABSRC1005026, ABSRC100502, ABSRC1005028 (slide no♀1449), ABSRC1005029, ABSRC1005030, ABSRC1005031, ABSRC1005032, ABSRC1005033, ABSRC1005034, ABSRC1005035, ABSRC1005036; occurrenceID: C3FF00AB-C126-5727-8057-CC4429D2FD47; **Location:** country: Kenya; locality: Rift Valley, Samburu Nat.Res. near Uaso Nyiro river, Intrepid Camp; verbatimElevation: 910 m; verbatimLatitude: 0°34’34,8”; verbatimLongitude: 37°39’36”; **Event:** samplingEffort: Black/MV lights; eventDate: 13-14-XII-2002
- **Type status:** Other material. **Occurrence:** catalogNumber: ABSRC1005019; recordedBy: K. Maes; individualCount: 5; sex: male; lifeStage: adult; disposition: ABSRC; otherCatalogNumbers: ABSRC1005022, ABSRC1005037, ABSRC1005038, ABSRC1005047 (slide no ♂994); occurrenceID: 2A876B0C-22AE-5620-AC83-D142EC84B568; **Location:** country: Kenya; locality: Rift Valley, Laikipia: Mpala Ranch near Nanyuki; verbatimElevation: 1692 m; verbatimLatitude: 00°17’36’’; verbatimLongitude: 36°53’55’’; **Event:** samplingEffort: Black/MV lights; eventDate: 16-IV-2002
- **Type status:** Other material. **Occurrence:** catalogNumber: ABSRC1005020; recordedBy: K. Maes; individualCount: 1; sex: female; lifeStage: adult; disposition: ABSRC; occurrenceID: 42901D80-92D8-545B-9CAE-A09CEC1166A7; **Location:** country: Kenya; locality: Rift Valley, Mathews Range, near Kitich Camp; verbatimElevation: 1360 m; verbatimLatitude: 01°13’47.1”; verbatimLongitude: 37°17’56.5”; **Event:** samplingEffort: Black/MV lights; eventDate: 9-10-XII-2002
- **Type status:** Other material. **Occurrence:** catalogNumber: ABSRC1005039; recordedBy: K. Maes; individualCount: 3; sex: male, female; lifeStage: adult; disposition: ABSRC; otherCatalogNumbers: ABSRC1005043, ABSRC1005044; occurrenceID: 8BEA1C14-75E3-5DCE-B6AA-F578029974C2; **Location:** country: Kenya; locality: Coast, Taita Discovery Centre, Makaramba; verbatimElevation: 495 m; verbatimLatitude: 3°40’22”; verbatimLongitude: 38°45’44”; **Event:** samplingEffort: Black/MV lights; eventDate: 3-VI-2000
- **Type status:** Other material. **Occurrence:** catalogNumber: ABSRC1005045; recordedBy: K. Maes; individualCount: 1; sex: female; lifeStage: adult; disposition: ABSRC; occurrenceID: A550F09B-4100-538F-B299-63996074E2C8; **Location:** country: Kenya; locality: Coast, Taita Discovery Centre, Makaramba; verbatimElevation: 495 m; verbatimLatitude: 3°40’22”; verbatimLongitude: 38°45’44”; **Event:** samplingEffort: Black/MV lights; eventDate: 27-III-2002
- **Type status:** Other material. **Occurrence:** catalogNumber: ABSRC1005040; recordedBy: K. Maes; individualCount: 1; sex: male; lifeStage: adult; disposition: ABSRC; occurrenceID: 67FC7A01-8BED-5EBE-B4B7-F465AAB075CA; **Location:** country: Kenya; locality: Lake Baringo Country Club; verbatimElevation: 1050m; verbatimLatitude: 0°38’; verbatimLongitude: 35°05’; **Event:** samplingEffort: Mercury Vapor Light; eventDate: 5-VII-1999
- **Type status:** Other material. **Occurrence:** catalogNumber: ABSRC1005041; recordedBy: K.Maes & A.Powys; individualCount: 3; sex: male, female; lifeStage: adult; disposition: ABSRC; otherCatalogNumbers: ABSRC1005042, ABSRC1005046; occurrenceID: 2EB7D969-1AE7-5310-AC1B-06F83DF3AA68; **Location:** country: Kenya; locality: Eastern, Meru N.P. Bwatherongi Camp Site; verbatimElevation: 620 m; verbatimLatitude: 00°09’57.9”; verbatimLongitude: 38°12’27.4”; **Event:** samplingEffort: Black/MV lights; eventDate: 30-31-XII-2001
- **Type status:** Other material. **Occurrence:** catalogNumber: ABSRC1005048; recordedBy: S.E.Miller & T.M.Kuklenski; individualCount: 1; sex: unidentified; lifeStage: adult; disposition: ABSRC; occurrenceID: 541145C1-2DA6-5A59-A281-3DBDD448F8E4; **Location:** country: Kenya; locality: Laikipia Plateau, Mpala Reserach Centre; verbatimElevation: 1692 m; verbatimLatitude: 0°17' 34.7994"; verbatimLongitude: 36°53' 56.4"; **Event:** eventDate: 13-15-II-1999
- **Type status:** Other material. **Occurrence:** catalogNumber: ABSRC1005049; recordedBy: S.E.Miller & T.M.Kuklenski; individualCount: 1; sex: female; lifeStage: adult; disposition: ABSRC; occurrenceID: C080BD7F-9E81-5EFB-B24A-D97DCF2FEFC6; **Location:** country: Kenya; locality: Laikipia Plateau, Mpala Reserach Centre; verbatimElevation: 1692 m; verbatimLatitude: 0°17' 34.7994"; verbatimLongitude: 36°53' 56.4"; **Event:** eventDate: 23-25-V-1998
- **Type status:** Other material. **Occurrence:** catalogNumber: ABSRC1005050 (slide no ♂880); recordedBy: S.E.Miller & T.M.Kuklenski; individualCount: 1; sex: male; lifeStage: adult; occurrenceID: 3B80ED44-7BB7-57BE-BE5D-28D5BD817B3D; **Location:** country: Kenya; locality: Laikipia Plateau, Mpala Reserach Centre; verbatimElevation: 1692 m; verbatimLatitude: 0°17' 34.7994"; verbatimLongitude: 36° 53' 56.4"; **Event:** eventDate: 14-16-XI-1998

#### Description

##### External characters

**Re-description**- Wingspan 11.8–15.1mm. Fore-wing length 7.0–7.7 mm. Head: Antenna yellowish-brown in the flagellum, greyish-white in the ciliae in both sexes. Frons and vertex yellowish. Labial palpus porrect, equal in length with the diameter of the eye, 1.4 times longer than wide (n = 4), scaling greyish-brown, interspersed with a small patch of white scales basally in the first segment in both sexes. Maxillary palpus obliquely upturned, length relative to the labial palpus 0.3 (n = 4), sporadically covered with greyish scales in segments 1 and 2 in both sexes. Thorax: Dorsum with yellowish scaling, venter greyish-white. Fore-wing ground of the upper side yellowish, with an oblique reddish-brown postmedial line running from the costa to the middle of the anal border, which is strongly diffuse to evanescent. Costa red-scaled from the base to the medial area. Termen and fringe reddish. Hind-wing ground of the upper side yellowish-grey. Abdomen: Venter greyish-white, dorsum yellowish in both sexes (Hampson (1913b), Figs 1b, 2b).

##### Male genitalia

Uncus very short, broad, constant in width, ratio length/width 1.4 (n = 3), apex tip rounded. Subscaphium absent. Tegumen shoulders medially broadened, projected. Transtilla widely separated, projected, transtillum inferior well developed. Valva with the basal costa inflated, basally strongly broadened, constricted in the posterior end, apex obliquely rounded towards the costa, sella rounded at both ends, medially constricted, posterior half dilated, anterior end spiculose. Transition from the basal to the post-basal sacculus distinct, from a narrowed basal sacculus to a strongly dilated post-basal sacculus, post-basal sacculus with a strongly sclerotised dorsal ridge covered with small chaetae. Juxta strongly broadened in the base, arms oblong, slender, strongly sclerotised. Vinculum broadened, v-shaped, saccus small. Sclerotisation of the anellus marked with two multifid, denticulate sclerites. Phallus with the posterior portion broadened, vesica bare from cornuti, sclerotisation marked with a small spiculose patch, coecum membranous (Figs 3b, 4b).

##### Female genitalia

Posterior apophysis elongate, unprojected. Anterior apophysis with a triangular-shaped projection medially, length relative to the posterior apophysis 1.5 (n = 1). Ostium membranous. Antrum with two strongly sclerotised, kidney-shaped flanges. Ductus bursae bare from sclerotisation. Corpus bursae globular-shaped, rhomboid signum with the longitudinal axis 2.3 times as long as the transversal axis (n = 1), lateral processes with medial sclerites, longitudinal ends flattened, unprojected. Second (posterior) signum ring-shaped, spiculose (Fig. 5b, c, d).

#### Diagnosis

*E.diatoma* is externally related with the Oriental *Ecpyrrhorrhoepuralis* (South, 1901) and *Ecpyrrhorrhoebiaculeiformis* Zhang, Li & Wang, 2004 sharing with these two species the yellowish-brownish ground. However, *E.diatoma* is differentiated from these two species by the presence of a reddish-brown oblique postmedial line in the fore-wing, in the male genitalia by the sclerotisation of the anellus marked as two multifurcate sclerites, by the shape of the sella with medial constriction and by the flattened, v-shaped vinculum. Furthermore, *E.diatoma* shares with *E.diffusalis* the broadened and rounded uncus, in which the two species differ from the type species *Ecpyrrhorrhoerubiginalis* (Hübner, 1796) and from all the Oriental species covered by the revision in Xiang et al. (2022). The uncus in the latter species is elongate and lanceolate. *E.diatoma* and *E.diffusalis* share with the type species the elongate arms in the bifid juxta, the presence of sclerites in the anellus and the shape and direction of the sella.

#### Distribution

Afrotropical – Somalia, Kenya, South Africa (De Prins and De Prins 2025). **New to the Arabian Peninsula.**

#### Biology

The larva of specimen no ABSRC1004140 was reared on *Leucusneuflizeana* Courbon (Lamiaceae). Pupa: 23-XII-2012, emergence of the adult: 03-I-2013.

### Euclasta varii

Popescu-Gori & Constantinescu, 1973

8EFE52EF-9D0A-5784-A039-B4C95F3F5736

*Euclastavarii* Popescu-Gorj & Constantinescu, 1973. Popescu-Gorj and Constantinescu (1973): 393–397. Type locality: Malawi, Mt. Mulanje

#### Materials

- **Type status:** Other material. **Occurrence:** catalogNumber: 24KSMA003; recordedBy: Al Dhafer, H., Fadl, H., Abd Eldayem, M., El Torky, A., El Gharbawy, A., Soliman, A.; individualCount: 2; sex: female; lifeStage: adult; disposition: KSMA; otherCatalogNumbers: 24KSMA004; occurrenceID: FD6C2549-1BD7-532E-9461-DB6A59DB17A1; **Location:** country: Saudi Arabia; stateProvince: Al Baha; locality: Shada Al Ala; verbatimElevation: 1610 m; verbatimLatitude: 19°50'24.66’'; verbatimLongitude: 41°18'41.46''; **Event:** eventDate: 27-VII-2015
- **Type status:** Other material. **Occurrence:** catalogNumber: 25KSMA001; recordedBy: Al Dhafer, H., Fadl, H., Abd Eldayem, M., El Torky, A., El Gharbawy, A., Soliman, A.; individualCount: 1; sex: female; lifeStage: adult; disposition: KSMA; occurrenceID: 25953493-712B-5523-8ED9-0B67B4B070C6; **Location:** country: Saudi Arabia; stateProvince: Asir; locality: Raydah; verbatimElevation: 2820 m; verbatimLatitude: 18°11'53.04''; verbatimLongitude: 42°24'26.1''; **Event:** eventDate: 12-XII-2014
- **Type status:** Other material. **Occurrence:** catalogNumber: 25KSMA002; recordedBy: Al Dhafer, H., Fadl, H., Abd Eldayem, M., El Torky, A., El Gharbawy, A., Soliman, A.; individualCount: 1; sex: female; lifeStage: adult; disposition: KSMA; occurrenceID: 0F4E1E66-2E1E-5FF4-BE28-567B14092831; **Location:** country: Saudi Arabia; stateProvince: Asir; locality: Raydah; verbatimElevation: 1850 m; verbatimLatitude: 18°11'40.74''; verbatimLongitude: 42°24'41.46''; **Event:** eventDate: 05-III-2015
- **Type status:** Other material. **Occurrence:** catalogNumber: 25KSMA004; recordedBy: Al Dhafer, H., Fadl, H., Abd Eldayem, M., El Torky, A., El Gharbawy, A., Soliman, A.; individualCount: 1; sex: female; lifeStage: adult; disposition: KSMA; occurrenceID: C5D01033-84C6-501E-83BA-A71D6F9BDC5F; **Location:** country: Saudi Arabia; stateProvince: Asir; locality: Raydah; verbatimElevation: 1850 m; verbatimLatitude: 18°11'40.74''; verbatimLongitude: 42°24'41.46''; **Event:** eventDate: 31-VII-2015

#### Diagnosis

Wingspan 29.7–32.1 mm. Fore-wing length 14.8–15.3 mm (n = 5). Labial palpus short, medially widened, acuminate in the third segment. Fore-wing ground light brown. Cell divided into two halves by an uninterrupted darkish-brown line, the upper half brownish scaled, the inferior half white scaled. Ductus bursae in the female genitalia with the anterior half widened and sclerotised, posterior half tapered, membranous, slightly dilated near the distal end. Length of the colliculum relative to the antrum 60% (n = 5). Antrum with a plate-shaped sclerotisation medially. Uncus in the male genitalia elongate, apex pointed. Tuba analis strongly exceeding the uncus, subscaphium present. Vesica with an elongate, slender cornutus (Popescu-Gorj and Constantinescu (1973), Figs 1c, 6a, b).

#### Distribution

Palaearctic – Southern Europe, Northern Africa. Afrotropical – Angola, Botswana, Congo, Malawi, Mozambique, Senegal, Sierra Leone, South Africa, Tanzania, Western Sahara, Zambia, Zimbabwe. For the Arabian Peninsula, the species has been recorded from Yemen (Popescu-Gorj and Constantinescu 1973). **New to Saudi Arabia.**

### Euclasta warreni

Distant, 1892

EA38D343-F243-5ACC-A0C4-93E2C62A7B16

*Euclastawarreni* Distant, 1892. Distant (1892): 241. Type locality: South Africa, Transvaal, Pretoria

#### Materials

- **Type status:** Other material. **Occurrence:** catalogNumber: 20GP025; recordedBy: M. Seizmair; individualCount: 2; sex: male, female; lifeStage: adult; disposition: CMS; otherCatalogNumbers: 20GP027; occurrenceID: D4D0BAAA-E26A-5D99-A553-FD5CD23AE977; **Location:** country: Oman; stateProvince: Dhofar; locality: Jabal Samhan, Viewpoint; verbatimElevation: 1400 m; verbatimLatitude: 17°6'9.38"; verbatimLongitude: 54°41'53.12''; **Event:** eventDate: 23-XI-2019
- **Type status:** Other material. **Occurrence:** catalogNumber: 24KSMA005; recordedBy: Al Dhafer, H., Fadl, H., Abd Eldayem, M., El Torky, A., El Gharbawy, A., Soliman; individualCount: 1; sex: female; lifeStage: adult; disposition: KSMA; occurrenceID: 1EAB08D4-D977-5E3C-B98A-E896E7105153; **Location:** country: Saudi Arabia; stateProvince: Al Baha; locality: Shada Al Ala; verbatimElevation: 1560 m; verbatimLatitude: 19°50'19.74'’; verbatimLongitude: 41°18'36.24'’; **Event:** eventDate: 17-X-2014
- **Type status:** Other material. **Occurrence:** catalogNumber: 24KSMA010; recordedBy: Al Dhafer, H., Fadl, H., Abd Eldayem, M., El Torky, A., El Gharbawy, A., Soliman; individualCount: 1; sex: male; lifeStage: adult; disposition: KSMA; occurrenceID: 9A283C22-0913-559F-83D7-47CFF6A2D197; **Location:** country: Saudi Arabia; stateProvince: Al Baha; locality: Shada Al Ala; verbatimElevation: 1670 m; verbatimLatitude: 19°50'34.5''; verbatimLongitude: 41°18.691’; **Event:** eventDate: 27-VII-2015
- **Type status:** Other material. **Occurrence:** catalogNumber: 25KSMA003; recordedBy: Al Dhafer, H., Fadl, H., Abd Eldayem, M., El Torky, A., El Gharbawy, A., Soliman; individualCount: 1; sex: female; lifeStage: adult; disposition: KSMA; occurrenceID: D4F64F3D-84C4-5C42-958C-A1B5E25E1DC0; **Location:** country: Saudi Arabia; stateProvince: Asir; locality: Raydah; verbatimElevation: 1850 m; verbatimLatitude: 18°11'40.74''; verbatimLongitude: 42°24'41.46''; **Event:** eventDate: 05-III-2015
- **Type status:** Other material. **Occurrence:** catalogNumber: 25GP04; recordedBy: M. Seizmair, A. Ishag; individualCount: 1; sex: male; lifeStage: adult; disposition: CMS; occurrenceID: 2E156964-7360-5C09-AADB-4DDE62CB7730; **Location:** country: Saudi Arabia; stateProvince: Jizan; locality: Fayfa Mts, Al Kasha; verbatimElevation: 640 m; verbatimLatitude: 17°14' 30.3684"; verbatimLongitude: 43° 3' 41.0178"; **Event:** eventDate: 30-X-2024

#### Diagnosis

Wingspan 36.6– 37.0 mm (n = 3) in the males, 37. 1 - 37.6 mm (n = 3) in the females. Fore-wing length 16.9– 17.5 mm (n = 3) in the males, 17.6 - 18.3 mm (n = 3) in the females. Labial palpus elongate, distal end acuminate, basal segment white-scaled. Maxillary palpus with greyish-brown scaling laterally. Fore-wing discal cell suffused with white scales over its entire range, with an orbiform black posterior discal spot and an infracellular darkish brown marking in the medial area of the anal border. Lower angle of the cell with a brownish-black strike bordered by a white line. Fringe grey. Uncus in the male genitalia with the neck short, constant in width, twice as long as wide, the apex of globular shape, rounded. Tuba analis equal in length with the uncus. Valva narrowed towards the apex, costa inflated basally, convex medially, distal end with a process terminating shortly below the apex. Phallus elongate, posterior portion slightly widened, vesica with a short, slightly curved cornutus, the anterior end of which is dilated. Posterior ductus bursae in the female genitalia with a strongly sclerotised dilation, followed by a short, strongly sclerotised colliculum and a slightly sclerotised antrum of subrectangular shape. Signum with short lateral processes (Popescu-Gorj and Constantinescu 1977, Figs 1d, 3c, 4c, 6c, d).

#### Distribution

Afrotropical – Ethiopia, Congo, Mali, South Africa, Zambia, Zimbabwe, Burundi, Kenya (Popescu-Gorj and Constantinescu 1973, Popescu-Gorj and Constantinescu 1977 and De Prins and De Prins 2025) . **New to the Arabian Peninsula.**

### Paschiodes postmediofusalis

(Seizmair, 2022) comb.n.

6D689A82-1676-5DE1-9634-543750A02422

*Pyraustamediofusalis* Seizmair, 2022. Seizmair (2022): 37-39. Type locality: Oman, Dhofar, Jebel Samhan, Viewpoint, 1400 m

#### Materials

- **Type status:** Other material. **Occurrence:** catalogNumber: 23GP011; recordedBy: M. Seizmair; individualCount: 1; sex: male; lifeStage: adult; disposition: CMS; occurrenceID: 080418D9-73AE-57B4-803A-DF8A26813167; **Location:** country: Saudi Arabia; stateProvince: Jizan; locality: Wadi Lajab, Khatwat Al Ain; verbatimElevation: 1277 m; verbatimLatitude: 17°35'20.34"; verbatimLongitude: 42°55'55.87"; **Event:** eventTime: 26-IX-2022

#### Diagnosis

Wingspan of the current record 24.1 mm. Fore-wing length of the current record 12.0 mm. The species complies with the internal differential character states of *Paschiodes* Hampson, 1913, namely the bifurcate chaetae of the uncus, the simple, strongly flattened sella and the sclerotisation of the vesica marked as a band of spines ranging over the entire length of the phallus. In the genus *Pyrausta* Hübner, 1825, the chaetae in the apical uncus are simple, the sella is of a distinguished linguiform shape, the sclerotisation of the vesica is marked as small cornuti variable in shape and number. The species is thus removed from *Pyrausta* Hübner, 1825 and transferred to *Paschiodes* Meyrick, 1890. The genus *Paschiodes* Meyrick, 1890 comprises five valid species, amongst which *P.postmediofusalis* comb. n. is unique in the greyish ground of the fore- and hind-wing upper side, the irroration by brownish scales in the fore-wing upperside, the yellowish tinge of the fore- and hind-wing costal areas, the flattened sella, the invaginated juxta, the elongate cornutus and the bulbous dilation in the posterior portion of the phallus (Seizmair 2022, Figs 3d, 4d) .

##### Remark

The only known record from Saudi Arabia is a strongly worn male specimen, which, however, could be clearly identified, by the male genitalia.

#### Distribution

Restricted to the Arabian Peninsula: Oman (Dhofar). **New to Saudi Arabia.**

## Supplementary Material

XML Treatment for Psammotis haematidea

XML Treatment for Ecpyrrhorrhoe diatoma

XML Treatment for Euclasta varii

XML Treatment for Euclasta warreni

XML Treatment for Paschiodes postmediofusalis

## Figures and Tables

**Figure 1a.**
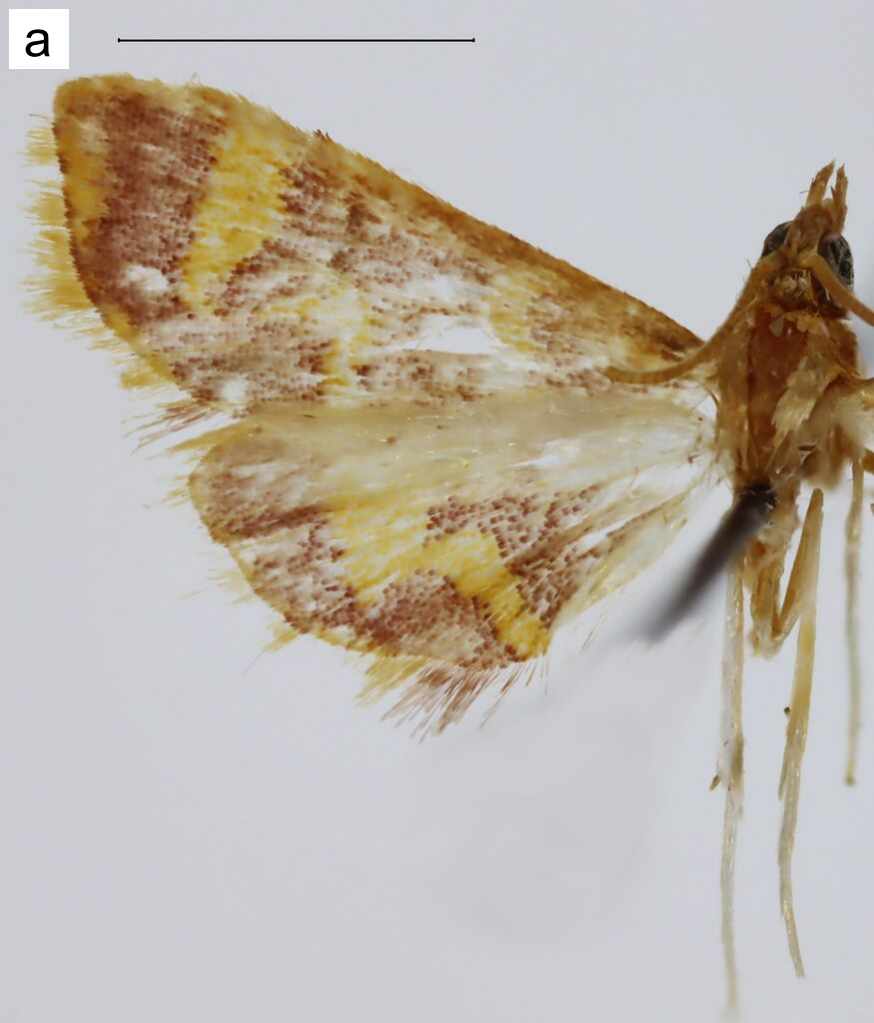
*P.haematidea*, slide no. 24GP028;

**Figure 1b.**
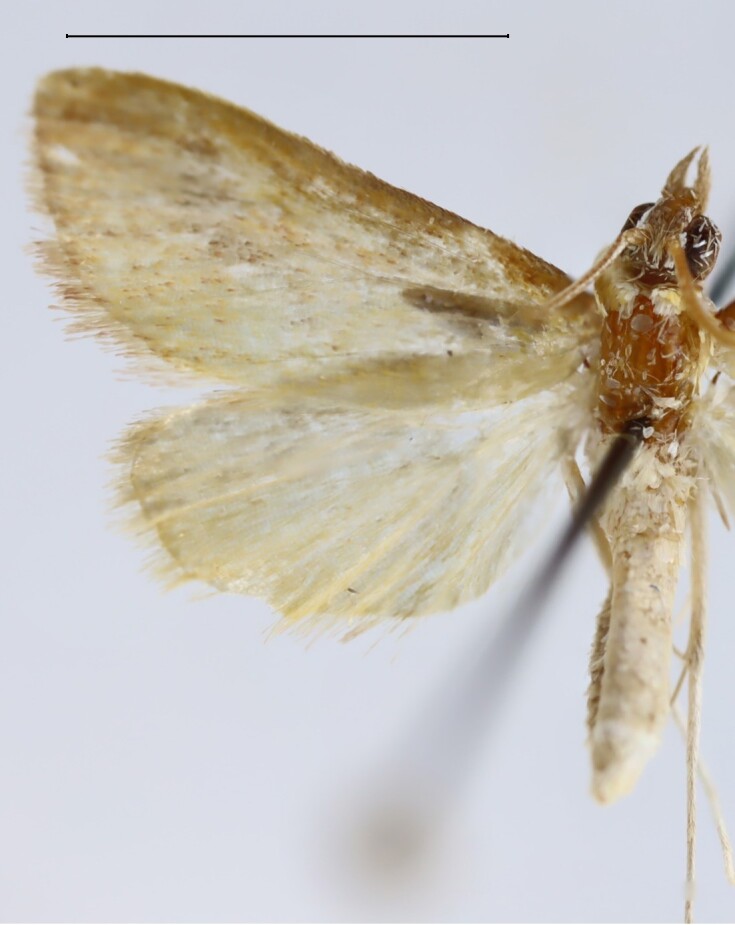
*E.diatoma*, slide no. 24GP032;

**Figure 1c.**
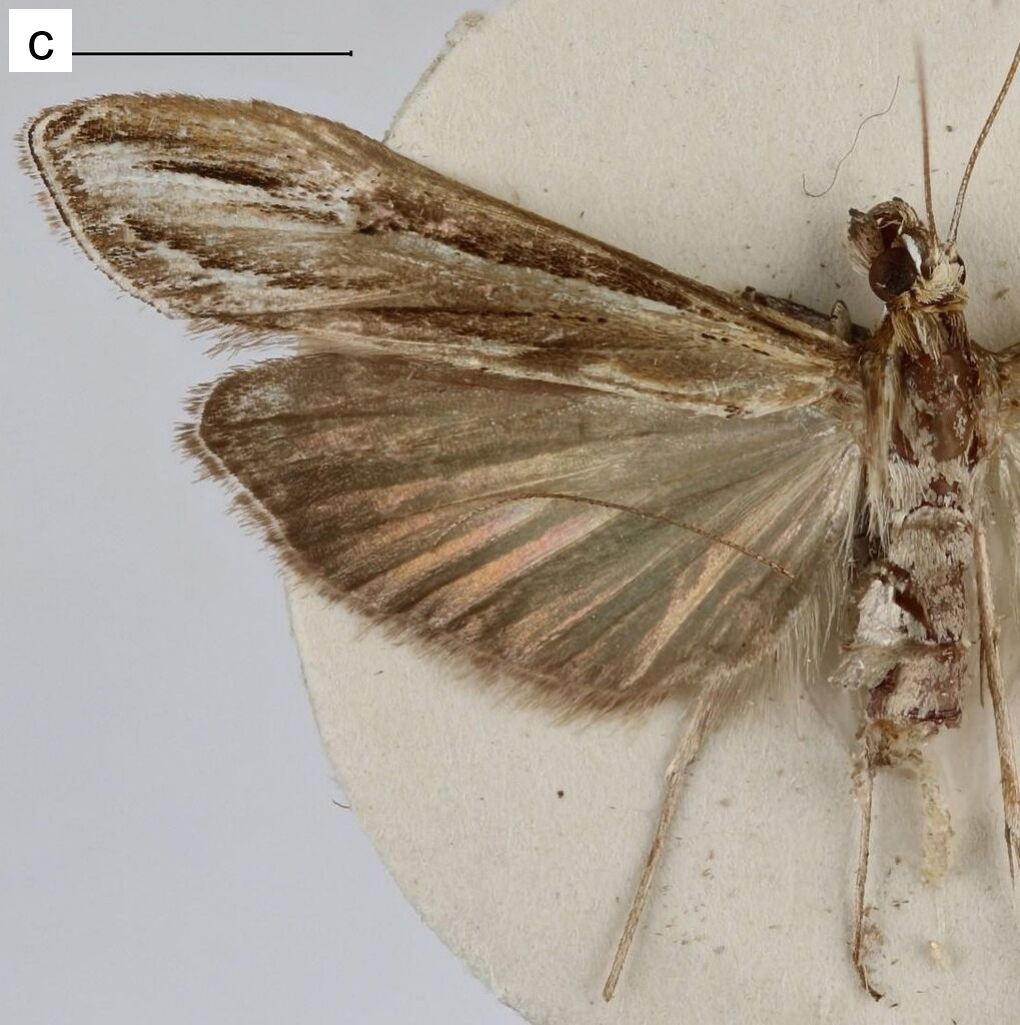
*E.varii*, slide no. 25KSMA001;

**Figure 1d.**
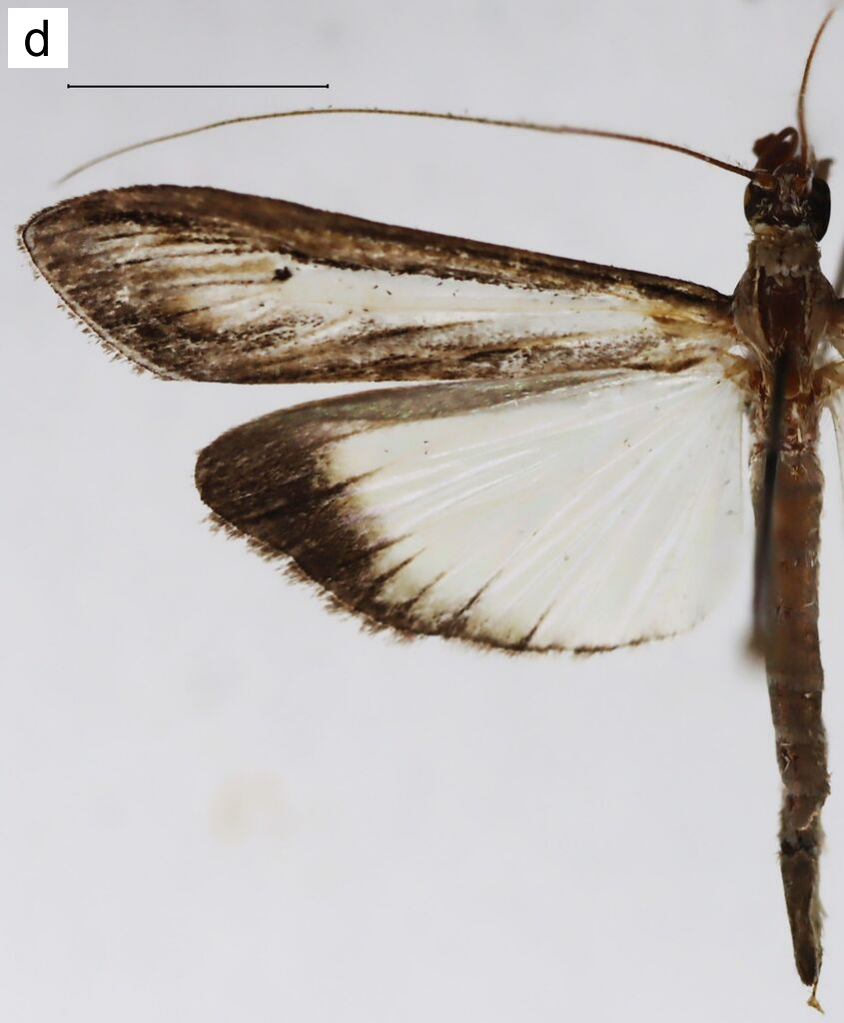
*E.warreni*, slide no. 25GP004.

**Figure 2a.**
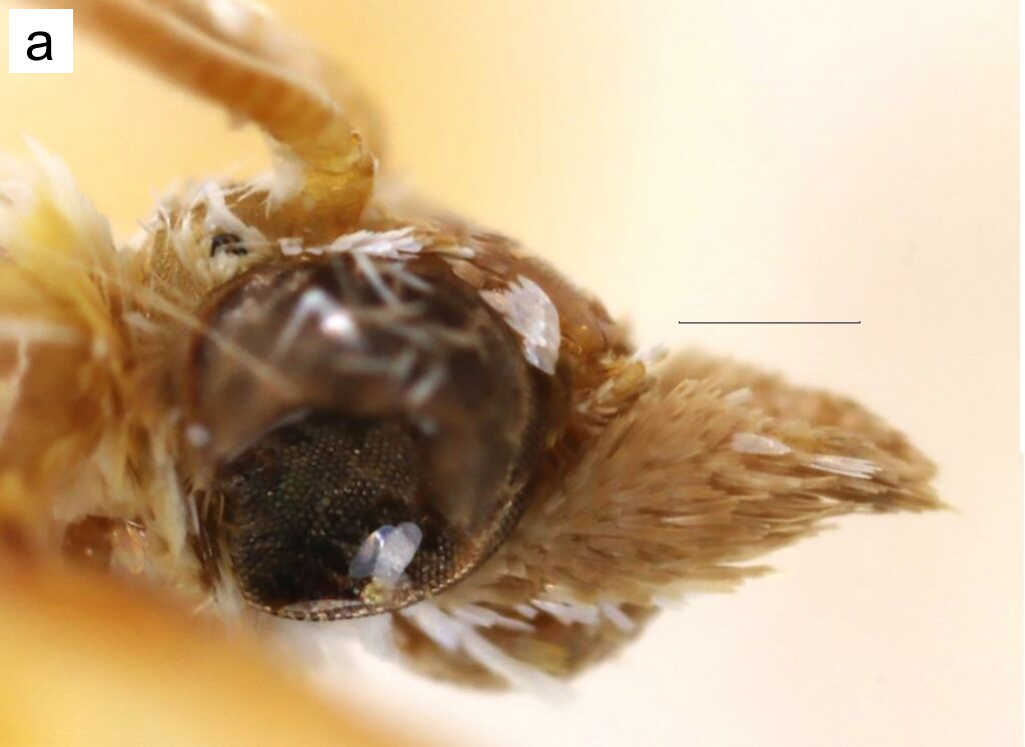
*P.haematidea*, slide no 24GP028;

**Figure 2b.**
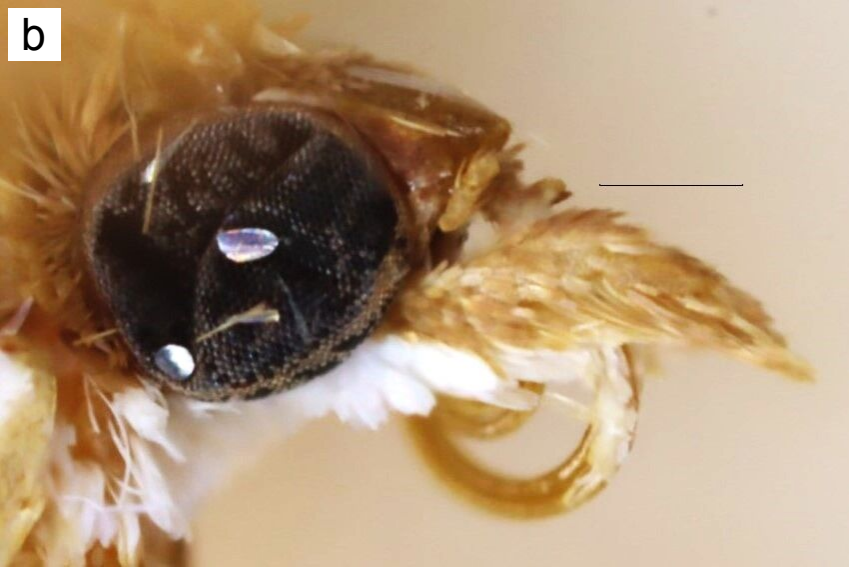
*E.diatoma*, slide no. 24GP034.

**Figure 3a.**
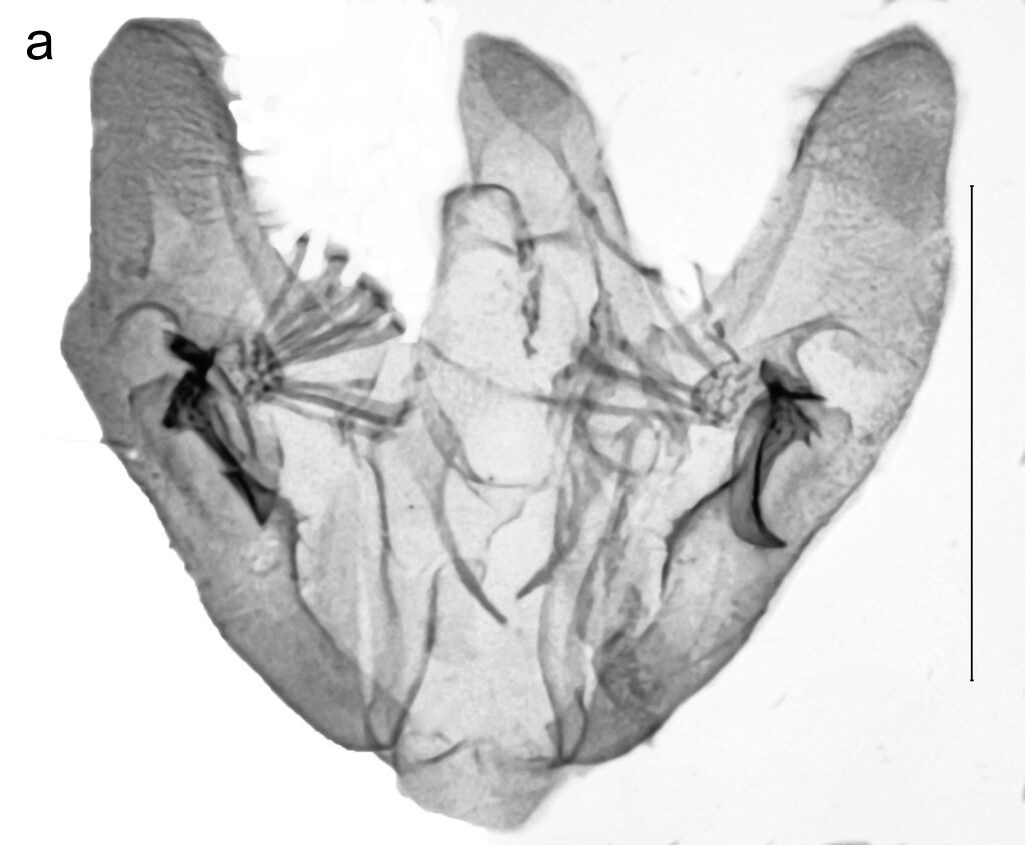
*P.haematidea*, slide no 24GP028, juxta omitted;

**Figure 3b.**
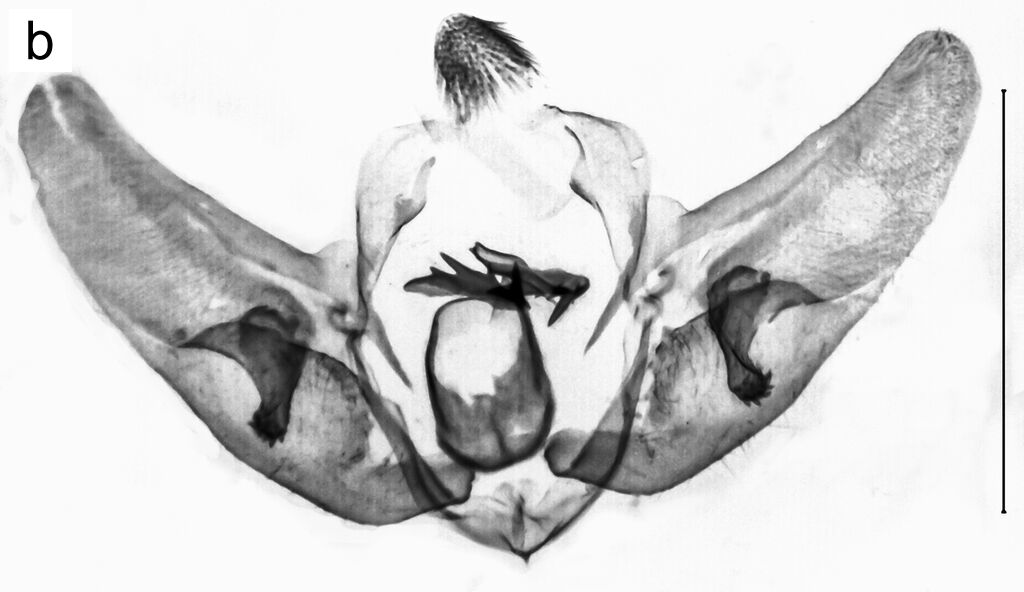
*E.diatoma*, slide no 24GP034;

**Figure 3c.**
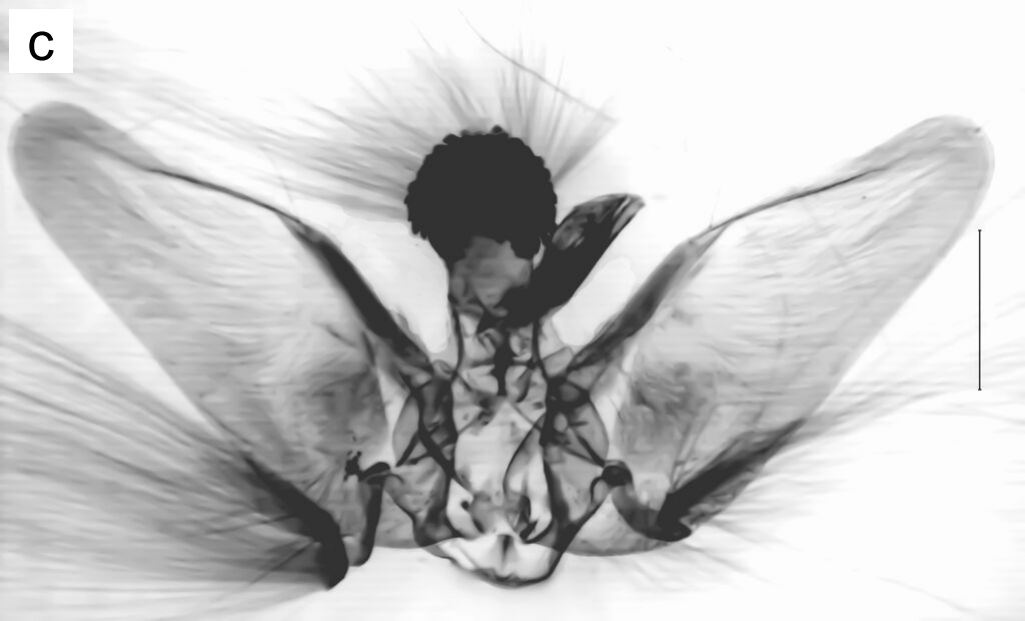
*E.warreni*, slide no 25GP004;

**Figure 3d.**
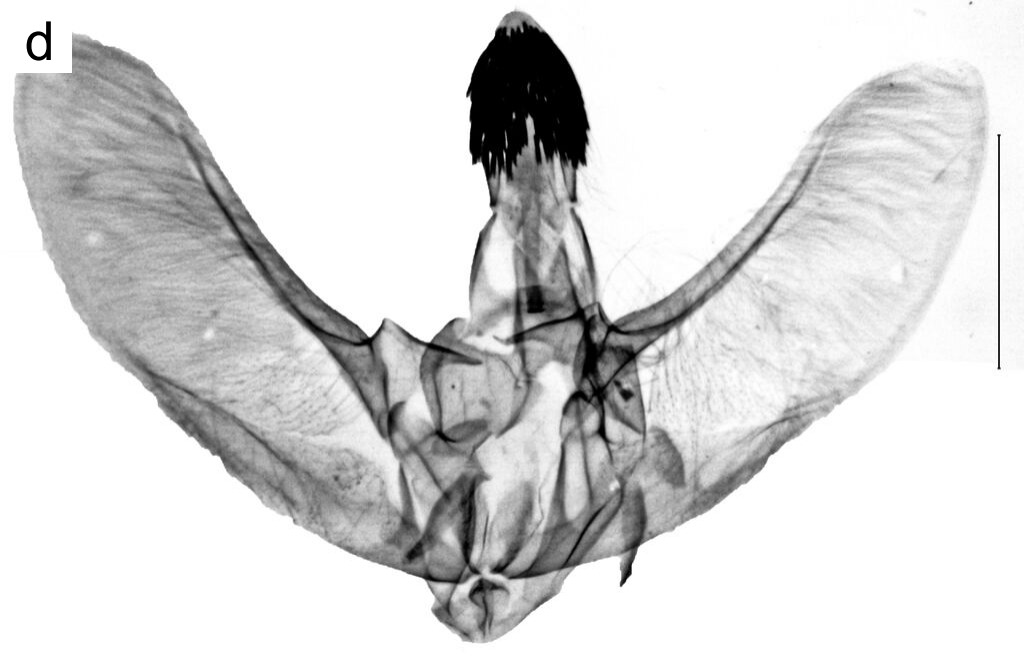
*P.postmediofusalis* comb. n., slide no 23GP011.

**Figure 4a.**
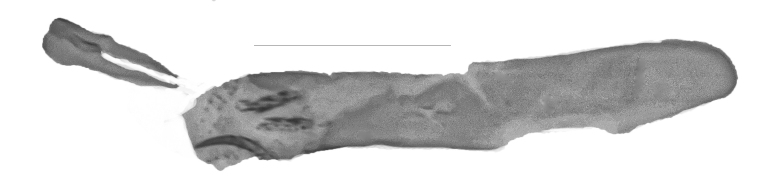
*P.haematidea*, slide no 24GP028, with juxta;

**Figure 4b.**
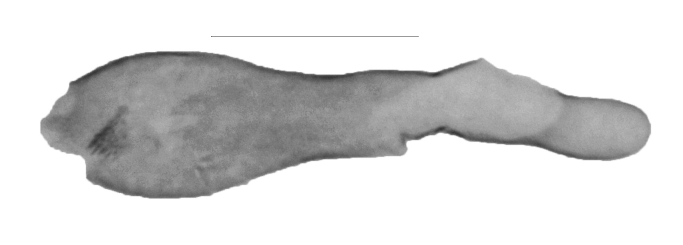
*E.diatoma*, slide no 22GP034;

**Figure 4c.**
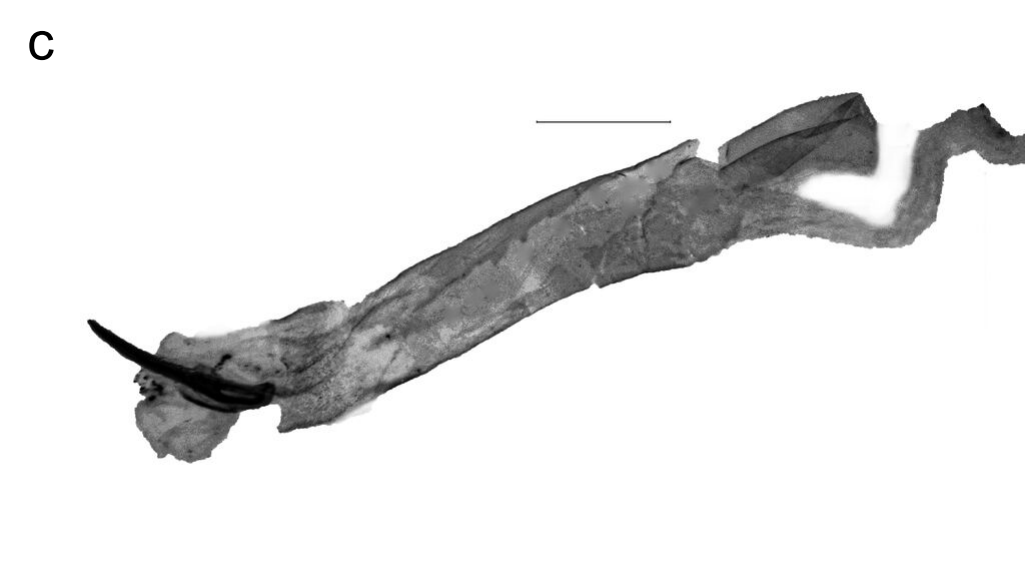
*E.warreni*, slide no 25GP004;

**Figure 4d.**
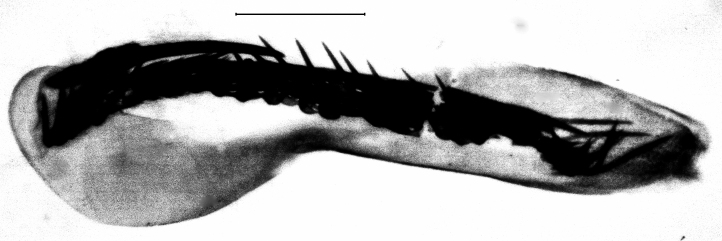
*P.postmediofusalis* comb. n., slide no 23GP011.

**Figure 5a.**
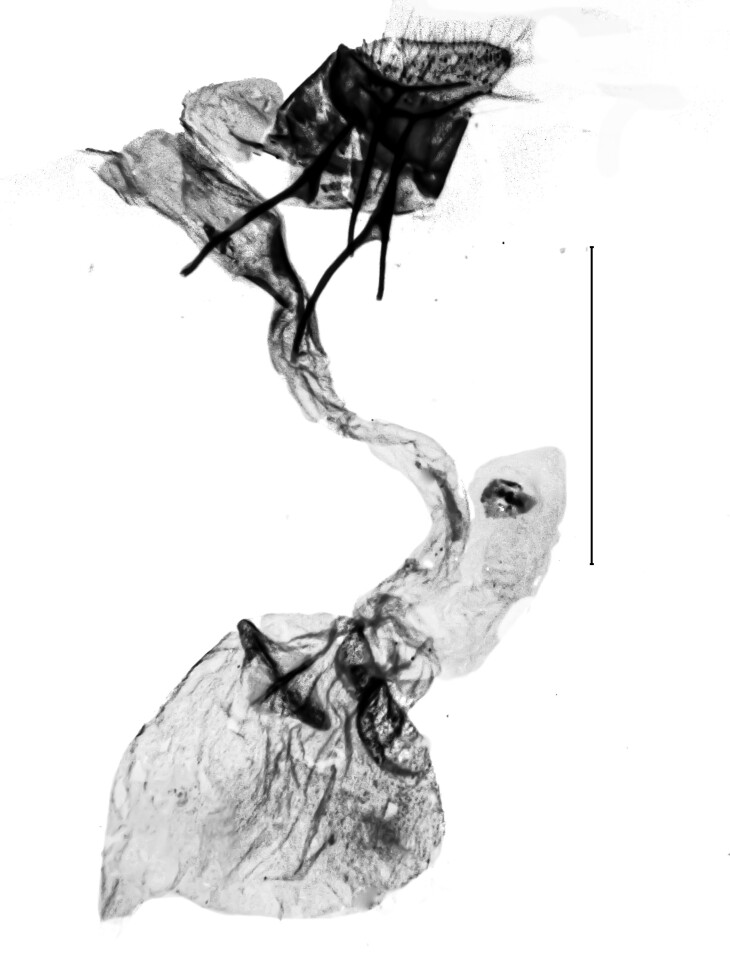
*P.haematidea*, slide no. 24GP027;

**Figure 5b.**
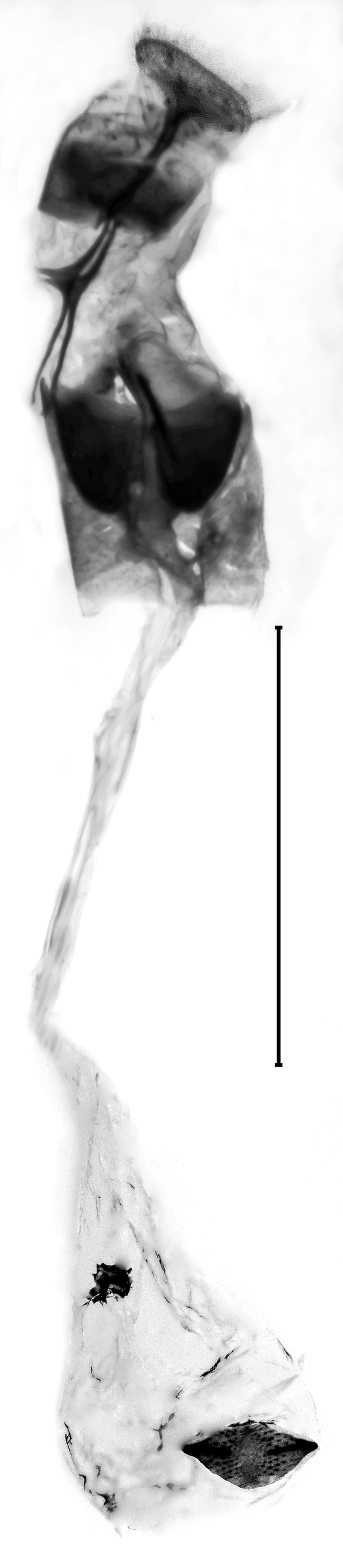
E.diatoma, slide no. 22GP066;

**Figure 5c.**
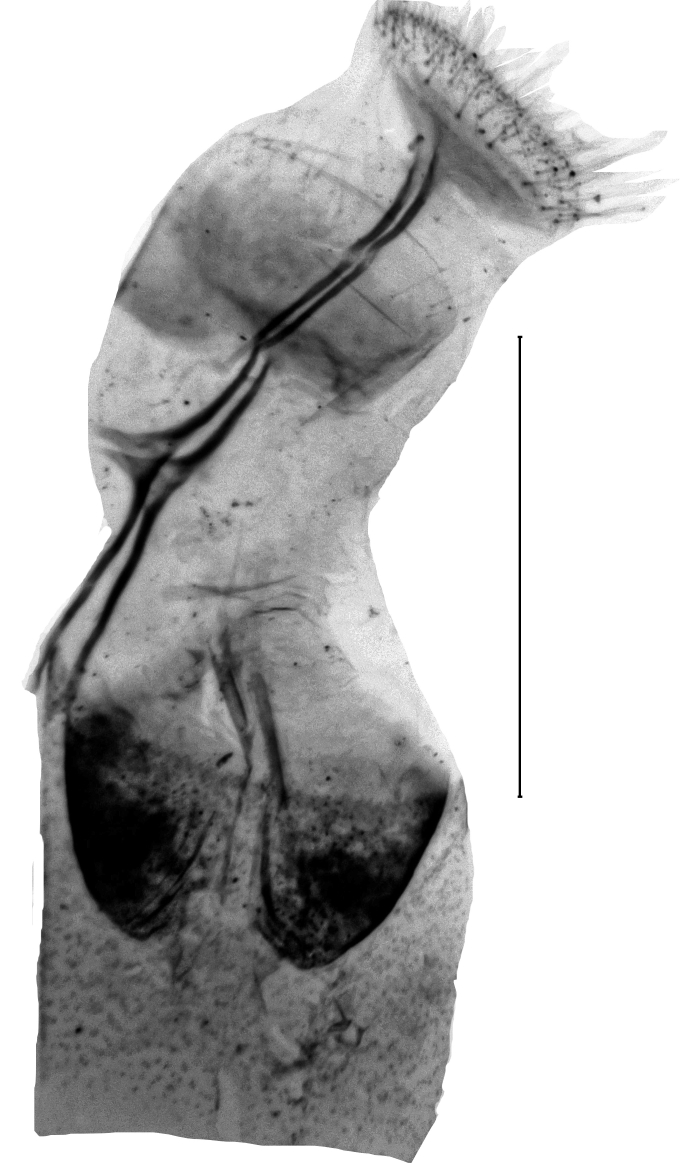
E.diatoma, slide no. 22GP066, close-up antrum, ovipositor;

**Figure 5d.**
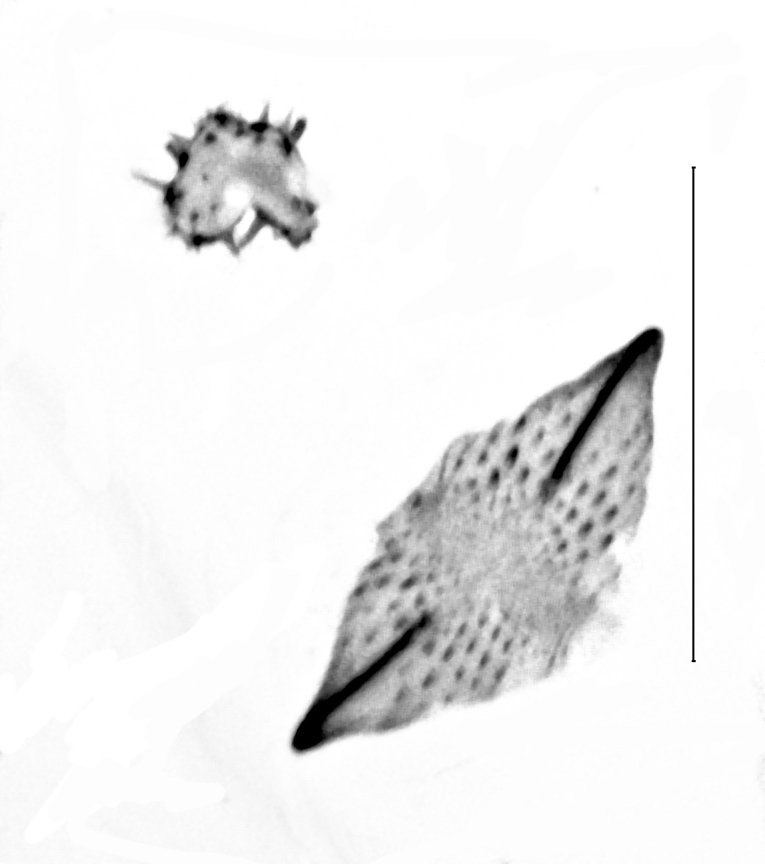
E.diatoma, slide no. 22GP066, close-up signa.

**Figure 6a.**
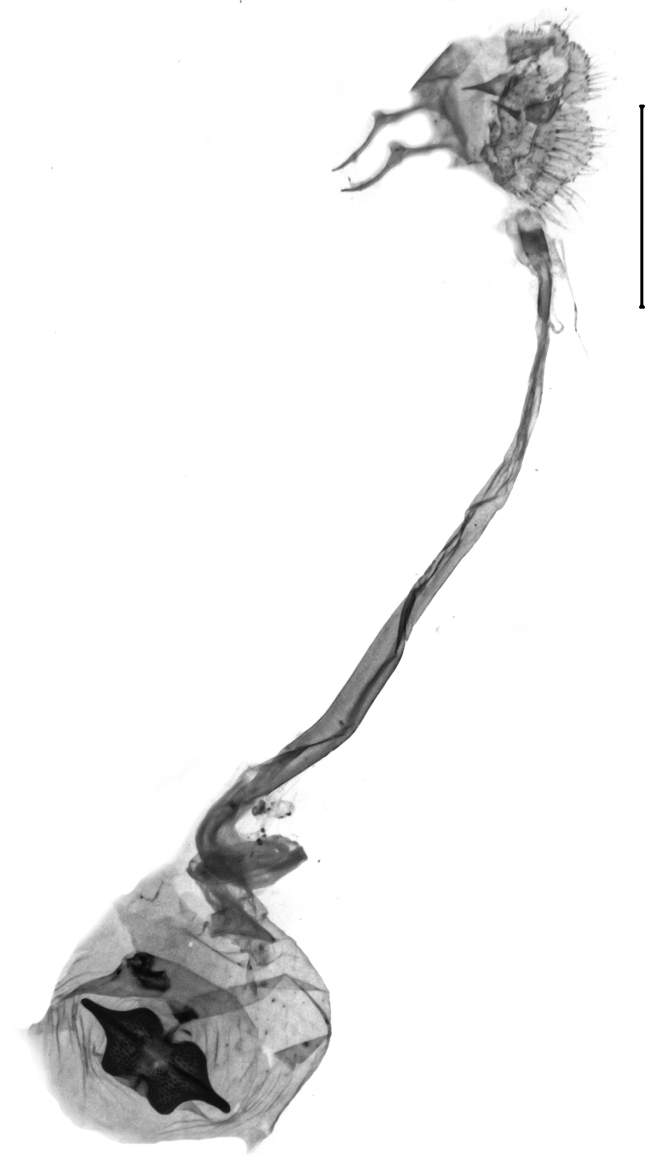
*E.varii*, slide no 24KSMA03;

**Figure 6b.**
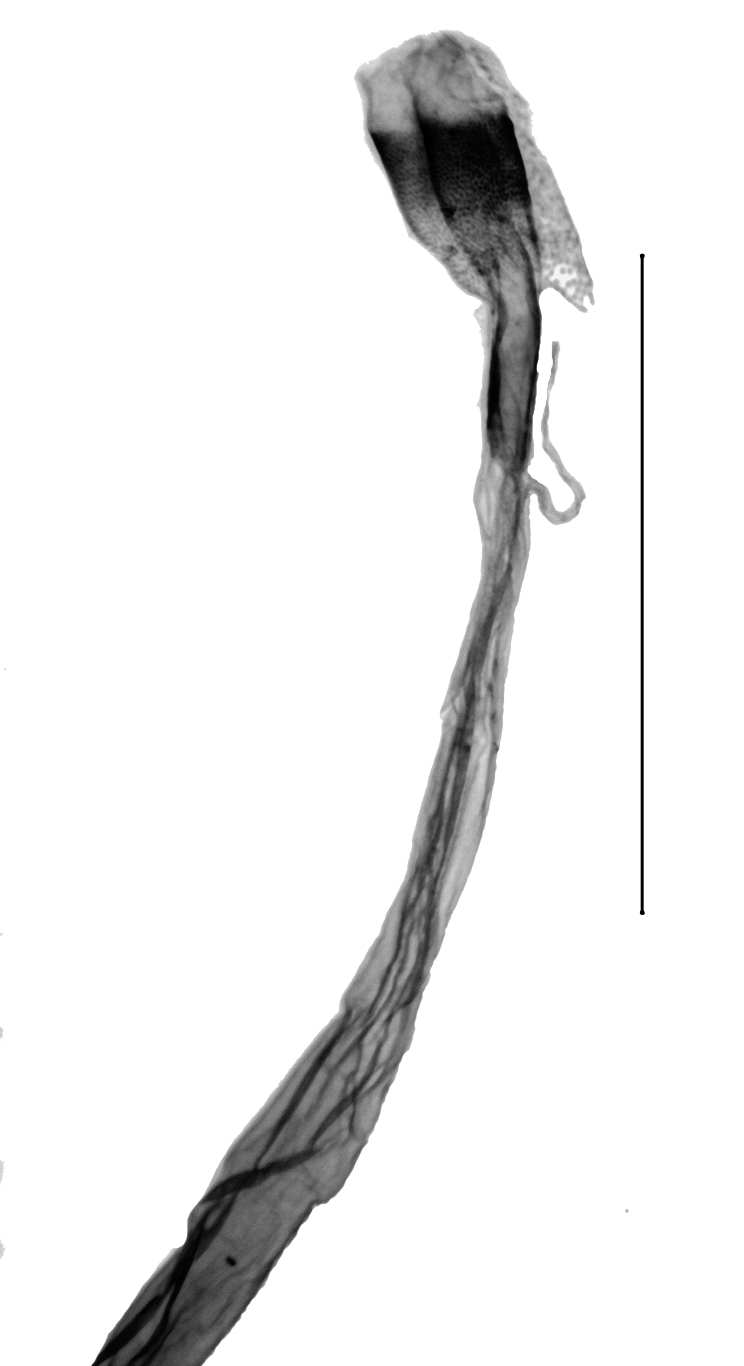
*E.varii*, slide no 24KSMA03, close-up posterior ductus bursae;

**Figure 6c.**
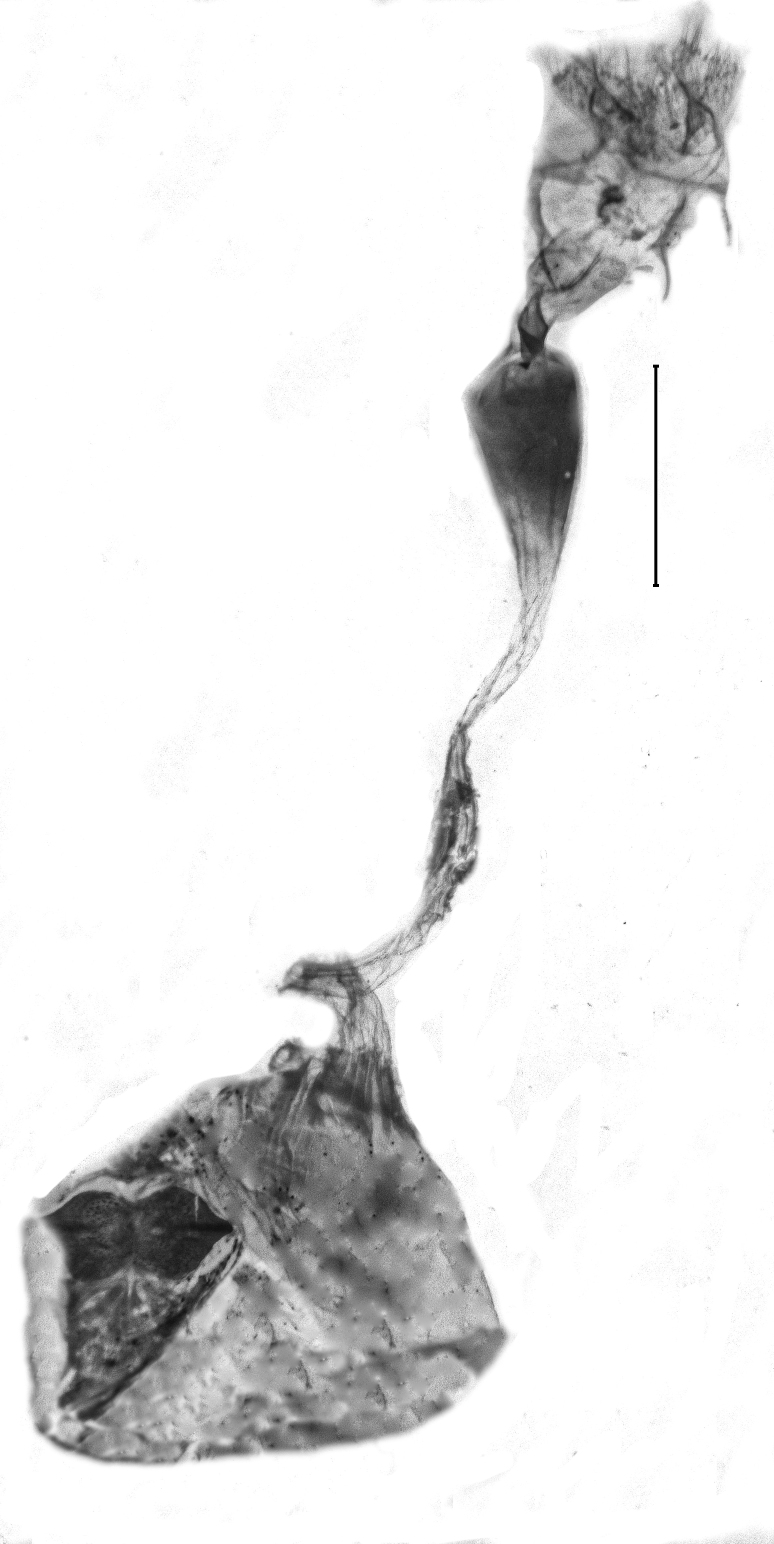
*E.warreni*, slide no. 24KSMA05;

**Figure 6d.**
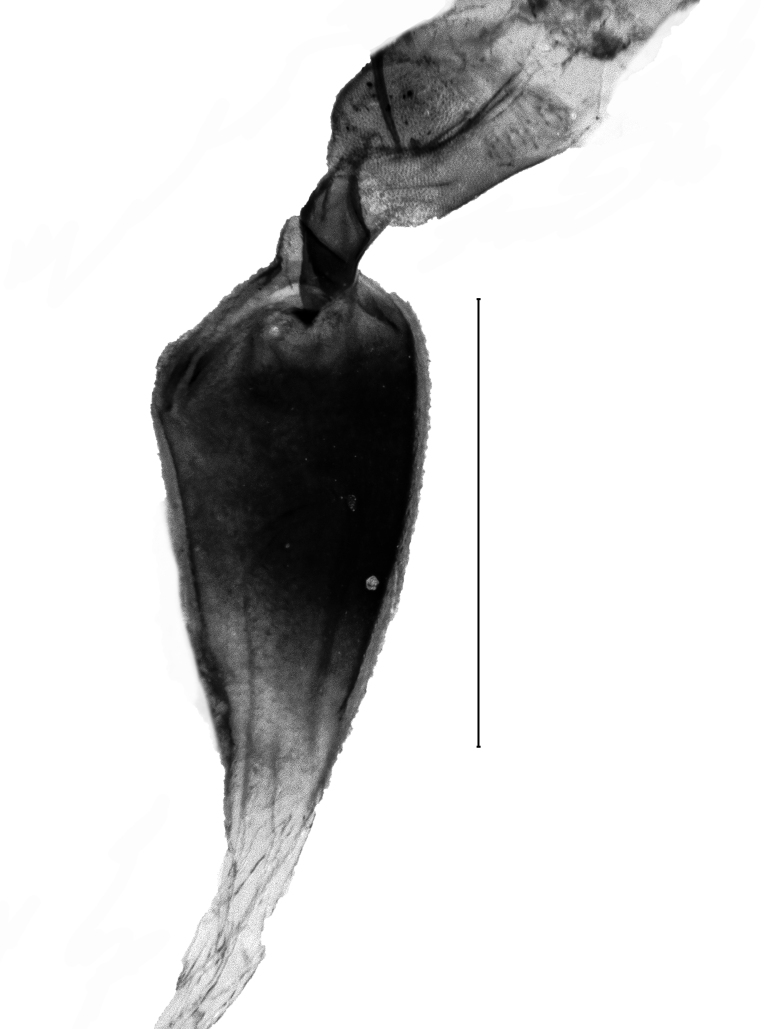
*E.warreni*, slide no. 24KSMA05, close-up posterior ductus bursae.

## References

[B12975497] Asselbergs J., van Harten A. (2008). Arthropod fauna of the UAE.

[B12975510] De Prins J., De Prins W. Afromoths online database of Afrotropical moth species (Lepidoptera). https://www.afromoths.net/.

[B13329210] Distant W. (1892). A naturalist in the Transvaal.

[B12975518] Guillermet C. (2009). Les Héterocères ou papillons de nuit, de l’île de la Réunion, Volume III, Familles des Pyralidae et Crambidae.

[B12975528] Hacker H. (2016). Systematic and illustrated catalogue of the Macroheterocera and Cossoidea Leach, [1815], Zygaenoidea Latreille, 1809, Thyridoidea Herrich-Schäffer, 1846 and Hyblaeoidea Hampson, 1903 of the Arabian Peninsula, with a survey of their distribution (Lepid.). Esperiana.

[B12975547] Hampson G. F. (1913). Descriptions of new Pyralidae of the subfamily Pyraustinae. Annals and Magazine of Natural History.

[B12975556] Hampson G. F. (1913). Descriptions of new Pyralidae of the subfamily Pyraustinae. Annals and Magazine of Natural History.

[B12975565] Hampson G. F. (1918). Descriptions of new Pyralidae of the subfamily Pyraustinae. Annals and Magazine of Natural History.

[B12975574] Hampson G. F. (1918). Descriptions of new Pyralidae of the subfamily Pyraustinae. Annals and Magazine of Natural History.

[B12975538] Hausmann A. (2006). The geometrid moth species from Yemen – with 50 new records for the country and description of 20 new taxa (Lepidoptera: Geometridae). Esperiana.

[B12975583] Landry B (2015). The Pyraustinae (Lepidoptera, Pyralidae s. l.) of the Galápagos Islands, Ecuador. Revue Suisse de Zoologie.

[B12975592] Maes K. V.N. (1995). A comparative morphological study of the adult Crambidae (Lepidoptera, Pyraloidea). Proceedings and Annals of the Belgian Entomological Royal Society.

[B12975601] Maes K. V.N. (2000). Revision of the genus *Paschiodes* Hampson (Lepidoptera: Pyraloidea: Crambidae: Pyraustinae). African Entomology.

[B12975610] Maes K. V.N (2001). *Herpetobotys* gen. n. with three new species from the Afrotropical region (Lepidoptera, Pyraloidea, Crambidae, Pyraustinae). Belgian Journal of Entomology.

[B12975619] Maes K. V.N. (2001). *Pseudognathobotys*, new genus with two new species of Pyraustinae (Lepidoptera, Pyraloidea, Crambidae). Belgian Journal of Entomology.

[B12975628] Maes K. V.N. (2001). *Cybalobotys* gen. n. with the description of three new species (Lepidoptera, Pyraloidea, Crambidae, Pyraustinae). Belgian Journal of Entomology.

[B12975637] Maes K. V.N. (2002). The genus *Crypsiptya* in Africa with the description of a new species and a world checklist of the genus species (Lepidoptera, Pyraloidea, Crambidae, Pyraustinae). Belgian Journal of Entomology.

[B12975646] Maes K. V.N. (2005). New species and new combinations in the Pyraustinae (Lepidoptera: Pyraloidea, Crambidae). Lambillionea.

[B12975655] Maes K. V.N. (2006). *Powysia* gen. n., a new genus of Pyraustinae for Eastern Africa (Lepidoptera, Pyraloidea, Crambidae). Belgian Journal of Entomology.

[B12975664] Maes K. V.N. (2006). *Thivolleo*, a new genus with two new species from Africa (Lepidoptera, Pyraloidea, Crambidae, Pyraustinae). Revue Suisse de Zoologie.

[B12975673] Maes K. V.N. (2009). A checklist of the *Pyrausta* species of Africa with description of a new species (Lepidoptera, Pyraloidea, Crambidae, Pyraustinae). Journal of Afrotropical Zoology.

[B12975682] Maes K. V.N. (2014). New taxa of the subfamily Pyraustinae from the Afrotropical region (Lepidoptera, Crambidae, Pyraustinae). Lambillionea.

[B12975691] Maes K. V.N. (2023). Studies on Crambidae III: New genus of Pyraustinae for the Afrotropical region (Lepidoptera: Pyraloidea: Crambidae: Pyraustinae). Metamorphosis.

[B12975700] Mally R., Hayden J., Neinhuis C., Jordal B. H., Nuss M. (2019). The phylogenetic systematics of Spilomelinae and Pyraustinae (Lepidoptera: Pyraloidea: Crambidae) inferred from DNA and morphology. Arthropod Systematics and Phylogeny.

[B12975710] Nuss M., Landry B., Mally R., Tränkner A., Vegliante F., Hayden J., Bauer F., Segerer A., Schouten R., Li H., Trofimova T., Solis M. A., De Prins J., Speidel W. Global Information System on Pyraloidea. https://www.pyraloidea.org/.

[B12975728] Pelham-Clinton E. C. (1977). Pyralidae from Oman: specimens in the Royal Scottish Museum, Edinburgh. Journal of Oman Studies, Special Report.

[B12991594] Poltavsky A. N., Sáfián S., Simonics G., Kravchenko V. D., Müller G. C. (2019). The Pyraloidea (Lepidoptera) fauna in the Liberian Nimba Mountains, West Africa, at the end of the dry season. Israel Journal of Entomology.

[B12975737] Popescu-Gorj A., Constantinescu A. (1973). New African species of *Euclasta* (Lepidoptera, Pyraustinae). Revue roumaine de biologie / Serie de zoologie.

[B12975746] Popescu-Gorj A., Constantinescu A. (1977). Revision of the genus *Euclasta* Lederer (Lepidoptera, Pyraustinae). A taxonomic and zoogeographic study. Travaux du Muséum d'Histoire Naturelle "Grigore Antipa".

[B12975764] Robinson G. (1976). The preparation of slides of Lepidoptera genitalia with special reference to the Microlepidoptera. Entomologist’s Gazette.

[B12975773] Rougeot P. C. (1984). Missions entomologiques en Ethiopie 1976–1982. Fascicule II. Mémoires du Muséum National d'Histoire Naturelle.

[B12991390] Seizmair M. (2021). S*cirpobotys xanthosomalis* gen. nov., sp. nov.—A new genus and species of the Pyraustinae (Lepidoptera, Crambidae) from the Arabian Peninsula. Trends in Entomology.

[B12991449] Seizmair M. (2022). The presence of the genus *Pyrausta* Schrank, 1802 (Lepidoptera, Crambidae, Pyraustinae) on the Arabian Peninsula - faunistic and taxonomic notes with description of a new species. Zoological and Entomological Letters.

[B12991468] Seizmair M. (2023). Contribution to the study of the Pyraustinae Meyrick, 1890 (Lepidoptera, Crambidae) on the Arabian Peninsula: A new species of *Psammotis* Hübner, 1825 from Saudi Arabia and new distributional data on four described species. Journal of Applied Entomologist.

[B12991487] Seizmair M. (2023). Contribution to the study of the Afrotropical Pyraustinae Meyrick, 1890 (Lepidoptera, Crambidae): Three new species from the southern Arabian Peninsula and distributional updates in the genera *Dysgrammodes* gen.n., *Pyrausta* Schrank, 1802 and *Anania* Hubner, 1823. Advances in Entomology.

[B12991496] Shaffer J. C., Munroe E. G. (2007). Crambidae of Aldabra Atoll (Lepidoptera: Pyraloidea). Tropical Lepidoptera.

[B12991505] Slamka F. (2013). Pyraloidea of Europe, Pyraustinae & Spilomelinae, Identification – Distribution – Habitat – Biology. Volume 3.

[B12991513] Vari L., Kroon D. M., Kruger M. (2002). Classification and checklist of the species of Lepidoptera recorded in Southern Africa.

[B12991521] Walsingham T., Hampson G. F. (1896). On moths collected at Aden and in Somaliland. Proceedings of the Zoological Society of London.

[B12991530] Xiang L., Chen K., Chen X., Duan Y., Zhang D. (2022). A revision of the genus *Ecpyrrhorrhoe* Hübner, 1825 from China based on morphology and molecular data, with descriptions of five new species (Lepidoptera, Crambidae, Pyraustinae). ZooKeys.

